# Doctors and “Doctors”

**Published:** 1890-07

**Authors:** 


### EDITORIAL DEPARTMENT.

F. E. DANIEL, M. D., EDITOR AND PUBLISHER.

THIS JOURNAL, by unanimous vote of the members, was made the Official Organ of the Austin District Medical Society.

COLLABORATORS:

*Dr. R. M. Swearingen, Austin.*
*Prof. B. E. Hadra, M. D., Galveston.*
*Prof. Geo. Cupples, M. D., San Antonio.*
*Dr. T. C. Osborn, Cleburne, Texas.*
*Dr E. J. Doering, Chicago.*
*Dr. E. J. Beall, Fort Worth.*
*Dr Odo Betz, Germany.*
*Prof. W. B. Rogers, M. D., Memphis.*
*Dr. J. W. McLaughlin, Austin.*
*Dr. T. J. Tyner, Austin.*
*Prof. J. F. Y. Paine, M. D., Galveston.*
*Dr. R. H. L. Bibb, Mexico.*
*Dr. T. J. Bennett. Austin.*
*Dr. Bat Smith, Wharton.*
*Dr. E. Meierhof, New York.*
*L. H. Luce, M. D., West Tisbury, Mass.*

### DOCTORS AND "DOCTORS."

Doctors may be likened unto verbs; they be regular, irregular and defective. We are speaking of "regular" doctors, for, ye medical profession, very properly, do not consider any man a doctor who has not a diploma from a regular college. With them, homeopaths, eclectics *et id om. gen.*, are not doctors at all; hence our remarks are confined to the "regular profession,"—taking no note of those who are confessedly irregular,—the *outside* quacks, except for comparison.

The "regulars" may be divided into the regular regular, the irregular regular, and the defective regular; then, again, some be both irregular and defective. Under the general head of the "regular profession" come all who can pass muster and become a member of the medical societies—State or local,—no matter by how close a skin of the teeth; all who can get in are understood to be "regular."

There be numerous—perhaps a majority of regulars, of the several degrees of regularity, who are not,—do not care to become members; the irregulars are those who could not if they would.

Now, doubtless, there are many comparatively good men who, having never graduated, could not, by any possibility, enter the ranks of the elect; doubtless, on the other hand there be many comparatively bad men who can and do get in; the difference between the extremes being about like that between a pig, a shoat and a hog. So, the line must be drawn somewhere, and it is drawn at the possession of a diploma! No matter how good a "doctor" may be as a practitioner, however well informed, successful and popular; however moral and correct, unless he have this one thing needful, the *open sesame*, he cannot come within the fold; no matter how ignorant and uncouth, nor how besotted, nor how mean and immoral, the possessor of a piece of sheepskin of the regulation sort—even *though it be one he cannot read*, can nearly always—(the exceptions are as the proverbial hen's teeth)—get some one to endorse his application, and can cross the line into the haven of regularity. The diploma, like charity, covers a multitude of faults. Once in, it is much more difficult to get him out; it cannot be done on anything "flimsy" or "puerile."

Let us diagram our idea, as Dr. Baldwin did the joke. D. for diploma, L. for learning or professional ability (more or less), C. for character.

Irregulars: (1) +L.+C.−D.<br>

(2) { +L.−C.−D. / +C.−L.−D. } (a pig)<br>

(4) −L.−C.−D. (a hog)

——— the line ———

Regulars: (3) −L.−C.+D. (a shoat)<br>

(2) { +L.−C.+D. or +C.−L.+D. }<br>

(1) +L.+C.+D. (the true physician.)

Dr. King, in his excellent little book,* has described the true physician; he possesses all the virtues. In our societies he predominates, it is to be hoped. Of course he is a "regular,"—a regular regular; but all regulars are not true physicians, un-

* "Tales of a Country Doctor," by Willis P. King, M. D., Kansas City; a most readable and good book.

fortunately. At a meeting he is easily distinguished. He is quiet, grave, gentlemanly, dignified; intelligence beams from his brow. He is there in the interest of scientific medicine; to see if he can learn something he did not know before, for he is willing to admit that there are some things he does not know, unlike certain other "regulars" to be presently mentioned; or perhaps, to impart, for the benefit of the profession, something in the life saving line that has come to him in his experience and observation. He shines in a crowd of regular, irregular and defective regulars, like a new "quarter," in a handful of coppers. God bless him and multiply him forever!

There be those, who would be thought ultra-regular (they are like the Indian's tree, "so straight it leaned over"), who pose as the very essence of regularity;—who quote the code with the same facility that they violate it in daily practice. These be hypocrites. They are keen observers of motes and beams in the eyes of others. In order to be thought regular in the extreme (the thief cries "stop thief"), these gents want to "run the machine." "We are the regular profession." Oftener than any other, one of these calls "Mr. President;" has something to say on every subject, and by emphasis and much gesticulation makes believe his remarks are full of meaning. He knows it all, and is there entirely in his own interest. If he has not been president he will be, if there be any virtue in electioneering and letter writing. That charity that covers a multitude of faults dwells not in his bosom. He knows all the faults and short-comings of his brethren, and conscious of his own moral taint, seeks by exaggeration in telling them around, to tarnish others; he may perhaps, whisper it behind his hand,—"h—sh! in confidence, you know,"—and under the seal of secrecy, writes or breathes insinuations and aspersions against a brother which, in his cowardly heart he knows to be false! Yet, he is a "regular." "Cæsar was an honorable man."

Then, there is the regular—ass. He is distinguished by his wise look and perpetual tooth pick; is affected in his speech, and as sanctimonious as he is deceitful. He knows it all. He listens to a paper with a "bored" air; smiles knowingly to himself, as if to say "Oh how perfectly familiar all that is to me." In days gone by he has covered all the ground; there is nothing for him to learn. He has a high opinion of himself, and would charge a rich corporation $5000 for an opinion, if he had the chance, and makes no bones in saying so; but, alas, for the chance!

Much irregularity crops out in the matter of advertising. Look in the great dailies of the State,—(and we have seen some pamphlets—even with a full moon face of the *soi disant* "specialist," published *before* admittance). In the daily are numerous quack advertisements; these are to be expected. There is the fellow who cures cancer without the knife. There is also, the advertisement of an eminent (?) regular, bordering so closely on the forbidden that were the name of a less regular-regular to it, it might be mistaken for a quack advertisement! Near the two may be a tradesman's cut, (as if to emphasize, not the difference, but the resemblance,) representing two of the small fry, very ragged from the rear aspect, which is presented, gazing in awe, far over the trouble waters of the broad Atlantic, and asking "what are the wild waves saying, brother?"

Now, the medical profession lay it to their hearts as a great grievance, that the public and the press will not discriminate;—that they regard *all* who call themselves "doctor," as one of a kind, and will see no difference between them; will not bow down and worship the regular profession as a superior set of mortals. How *can* they, when this style of advertising seems to be common property with all "schools," and is resorted to occasionally, rarely, we are glad to say, by the *known* elect, (?) when they see men of quackish proclivities and practices taken into the fold? How can they know who constitute the regular profession, or where to draw the line? Neither a newspaper nor the most astute public could, with a high power microscope detect any difference between these advertising regulars and the "other fellows;" the regular, having met the irregular on this common ground, the presumption is that they are *par nobile fratrum*. The former cannot go to the newspaper nor to the "astute public," and say: "Behold! here is my diploma; this let me into the State Association, despite my tricks; the other fellow hasn't any!" He cannot exhibit it, as he did to the Judicial Council; and yet, in the absence of a knowledge of its possession, how can they tell "which from t'other?" And how funny it must seem to disinterested parties, to the press and public, to see these fellows, an inside quack, an outside quack, seriously engaged in a game of Kettle and Pot! The profession, *on principle*, must back up their Kettle, although, perhaps, knowing it to be as dirty as the Pot; but the public can see no difference. The diploma being out of sight *they* have only appearances and past record to judge by—dull and indiscriminating public!

This is intended in nowise as a reflection on the large body of honest, even though humble workers in the ranks, the bone and sinew of the profession. They are code-respecting, modest and meritorious seekers after truth, respecting themselves and cherishing a high regard for the rights of others. A defective preliminary or literary education, while a great drawback, detracts nothing from their worth as practictioners of a noble profession, however it may affect their ability, provided they are sincere, and are actuated by the true principle that should govern every practitioner of rational medicine; but, who will deny that there is a large element of unworth in the organized profession, that leavens the loaf, as we have once before pointed out, and brings discredit on the whole? blatant, loud quacks, wolves in sheeps' clothing, men, whose character, in the communities where they have lived, will not bear the calcium light of investigation, yet whose chief delight is the defamation of a brother physician? Nor is this intended as a reflection on the Judicial Council. Those gentleman are honorable and high-minded, and are inspired by a jealous regard for the character of the Association; but they examine applications in the light in which they are presented, and doubtless are often imposed upon. We know of cases where they have been either deceived or satisfied by specious promises, and the anomaly exists in Texas of the State Association harboring members who had been black-balled by local societies; of admitting to membership one who had been expelled from a local Society for practicing on a Buchanan diploma.

Is there a remedy?

The mission of the JOURNAL, as often announced, is, or was the advancement, elevation and *purification* of the medical profession of Texas. Talk of "purification?" Vain hope! When at the fountain head, in the organized department, we have seen charges of *conduct unbecoming a gentleman and a physician*, backed by strong documentary evidence, *suppressed* "in the interest of harmony," or pronounced unworthy of serious consideration; and the accused, (doubtless out of gratitude) has ever after been the advocate and champion of "harmony;" carrying his zeal so far, in an underhanded way, as to *raise a row*, so to speak, and cause a serious split, which really threatened the integrity of the Association, *in order that there might be harmony!* Like Buck Fanshaw, "the peacablest man in Nevada," who "licked eleven Greasers in fifteen minutes, because they wouldn't be peacable!" (he "would *have* peace," according to Scotty Briggs, in Mark Twain's inimitable book,) this accused and condoned party was so bent on "harmony," that he would, without cause, depose "the best Secretary the Association ever had," according to his own admission; and in the attempt to do so, got up an ebullition of feeling among the members, all for the sake of "harmony!" Oh harmony, harmony! how many sins are committed in thy name!

It is of record that the Texas State Medical Association has outraged justice, robbed its own treasury, and compromised its reputation for intelligence—for the sake of "harmony." It has witnessed the vilest slanders by a member against a brother member of a neighboring State Association, which publication the said Association has, by official action, denounced as false and slanderous, and for the sake of "harmony," has taken no action in self-vindication, but stands to-day, committed by its silence, as tacitly endorsing the action of its member.

It had been hoped that in organization the remedy had been found; that by careful selection, a State Association might be made to represent exclusively the better element of the profession; but since Tom, Dick and Harry have been admitted, it has become almost as heterogenous as the profession at large, and separation and purification are no longer possible. The JOURNAL'S missson is gone, that part of it at least. To purify the profession of Texas, would be to do as McCarty did with his dog: cut his tail off *just back of the ears!* We give up the purification business; too many of our *big ones* would have to go with the tail part.

The remedy can apply only to the future. We must bear the ills we have, it is to be presumed, but hereafter a closer scrutiny into the character and standing of all applicants should be exercised. It is not alone a question of whether or not one has a diploma. The Associations of physicians are semi-social in their character, and unimpeachable moral character and unquestionable professional integrity should be the *sine qua non* to membership.

